# Mechanical and Antimicrobial Properties of Nanocellulose‐Reinforced Heat‐Cured Versus the Conventional Heat‐Cured Acrylic Resins: An In Vitro Study

**DOI:** 10.1155/ijod/4009010

**Published:** 2026-07-03

**Authors:** S. G. Padmapiriyaa, Nanditha Kumar M., Thippeswamy Honne Manjunathappa

**Affiliations:** ^1^ Department of Prosthodontics and Crown and Bridge, JSS Dental College and Hospital, JSS Academy of Higher Education and Research, Mysore, 570015, India, jssuni.edu.in; ^2^ Department of Public Health Dentistry, JSS Dental College and Hospital, JSS Academy of Higher Education and Research, Mysore, 570015, India, jssuni.edu.in

**Keywords:** antimicrobial property, flexural strength, heat-cure acrylic resin, impact strength, nanocellulose

## Abstract

**Background:**

Edentulism, the loss of natural teeth, affects mastication, speech, and esthetics. Dentures, usually made of heat‐cured acrylic resin, replace function and esthetics. Conventional heat‐cured acrylic resin—polymethyl methacrylate (PMMA), used for dentures is susceptible to fracture and fungal colonization by *Candida*, which can cause denture stomatitis. This study was done to investigate reinforcing PMMA with nanocellulose and its effect on the flexural strength, impact strength and antimicrobial properties of PMMA.

**Materials and Methods:**

A total of 90 heat‐cured acrylic resin specimens were fabricated using DPI Heat Cure acrylic resin (The Bombay Burmah Trading Corporation Ltd., India) with nanocellulose particles (Vedayukt India Pvt. Ltd., Jharkhand, India), incorporated. The samples were divided into three groups: control (conventional PMMA), 2.5% nanocellulose‐reinforced PMMA, and 5% nanocellulose‐reinforced PMMA. The specimens were evaluated for flexural strength, impact strength, and antifungal activity against *Candida albicans*. Statistical analysis was performed using one‐way ANOVA followed by Bonferroni post hoc test.

**Results:**

Flexural strength increased with nanocellulose concentration, but the change was not statistically significant (*p* = 0.095). In contrast, impact strength showed a significant, dose‐dependent improvement (*p*  < 0.001), with the 5% group being substantially stronger than the 2.5% group and the control group. No antifungal activity was observed in any group.

**Conclusions:**

Within the limitations of this in vitro study, nanocellulose reinforcement of PMMA produced a significant, concentration‐dependent increase in impact strength, with a nonsignificant improvement in flexural strength. The findings suggest that 5% nanocellulose reinforcement may enhance denture impact resistance and reduce fracture risk. No antifungal activity against *Candida albicans* was observed.

## 1. Introduction

Edentulism refers to the absence of some or all natural teeth and is categorized as total or partial. Primary factors include periodontal disease, dental caries, trauma, and hereditary influences. Tooth loss affects articulation, esthetics, and mastication, resulting in a diminished quality of life [1]. Dentures serve as a prevalent rehabilitative approach, facilitating the restoration of function, augmentation of self‐esteem, and enhancement of esthetics [2].

Prosthetic rehabilitation involves either fixed or removable dentures, with the selection based on the patient’s needs, available bone support, and medical condition. Removable dentures typically consist of artificial teeth mounted on a supporting base, which is usually fabricated from acrylic resin or metal [3].

The denture base supports prosthetic teeth and rests on oral tissues and is used in complete or partial dentures. Polymethyl methacrylate (PMMA) is the most common material used due to its color compatibility, biocompatibility, dimensional stability, cost‐effectiveness, and resistance to oral fluids [4, 5]. PMMA is widely used in dentistry for denture bases, artificial teeth, obturators, orthodontic retainers, provisional crowns, occlusal splints, and study models [6].

According to ADA Specification Number 12, denture base polymers are classified by their polymerization method into heat‐cured PMMA, auto‐polymerizing PMMA, and thermoplastic types. ISO 20795‐1:2013 further introduced light‐activated and microwave‐cured polymers. Heat‐cured acrylic resin remains the most popular due to its light weight, affordability, ease of fabrication, and color matching, despite drawbacks like low thermal conductivity and brittleness [5]. PMMA must have strong mechanical properties to resist mastication forces, but denture fractures, often from flexural or impact fatigue, are still common [6, 7].

To improve mechanical properties, researchers have incorporated reinforcements like glass fiber, polyethylene fiber, metal wire, carbon fiber, and nanoparticles into denture base resins. The effectiveness of nanoparticles depends on their size, shape, surface area, concentration, and dispersion. Plant‐based nanoparticles, in particular, offer advantages like biodegradability, biocompatibility, and enhanced material properties.

Recent research has focused on improving dental materials, with growing attention to environmental sustainability [8]. Nanocellulose, derived from sources like wood, plants, and agricultural waste, is inexpensive, biocompatible, and ecofriendly and offers excellent mechanical, chemical, and optical properties. Its durability, anisotropic structure, and nontoxic nature make it highly valuable in material and biomedical sciences [9].

Removable prostheses can alter oral ecology, increasing bacterial and fungal populations. Denture base resins, especially heat‐cured PMMA, commonly harbor organisms like *Candida albicans* and *Candida glabrata* [10–12]. Biofilm formation by Candida species is a major cause of denture stomatitis.

Hence, this study incorporated nanocellulose in varying proportions into heat‐cured acrylic resin to improve its mechanical and antimicrobial properties, and compared the results with conventional heat‐cured acrylic resin.

## 2. Materials and Methods

An in vitro experimental study was conducted to evaluate the mechanical and antimicrobial properties of nanocellulose‐reinforced heat‐cured acrylic resin under standardized laboratory conditions.

### 2.1. Materials

The materials used in this study included heat‐polymerized acrylic resin (DPI Heat Cure, The Bombay Burmah Trading Corporation Ltd., India), nanocellulose particles (Vedayukt India Pvt. Ltd., Jharkhand, India), dental stone (Kaldent; Kalabhai Karson Pvt. Ltd., Mumbai, India), and a sodium alginate‐based separating medium (Cold Mold Seal, Dental Products of India, Mumbai, India).

### 2.2. Specimen Preparation

A total of 90 specimens were fabricated and allocated to three experimental evaluations: flexural strength, impact strength, and antimicrobial activity (*n* = 30 per group). For each evaluation, specimens were randomly divided into three groups (*n* = 10 each):

• Group I (control): conventional heat‐cured PMMA.
• Group II: PMMA‐reinforced with 2.5 wt% nanocellulose.
• Group III: PMMA‐reinforced with 5 wt% nanocellulose.

### 2.3. Preparation of Nanocellulose‐Reinforced PMMA

Nanocellulose particles were incorporated into the acrylic polymer powder at concentrations of 2.5 wt% and 5 wt%. The required quantities were measured using a calibrated analytical balance (accuracy: ±0.001 g). The nanoparticles and polymer powder were blended manually using a mortar and pestle.

To ensure uniform dispersion and minimize particle agglomeration, the geometric dilution technique was employed [13].

### 2.4. Assessment of Flexural Strength

#### 2.4.1. Fabrication of Test Specimens

The test specimens were fabricated to a size of 65 mm × 10 mm × 3.3 mm, following the guidelines of ISO 20795‐1:2013. Wax patterns were prepared using standardized stainless‐steel molds corresponding to the required specimen dimensions. Flasking was carried out using a dental stone, followed by dewaxing in a thermostatically controlled water bath. A separating medium (alginate‐based solution, Cold Mold Seal, Dental products of India, India) was painted uniformly to the mold surfaces. Three distinct formulations of heat‐cure acrylic resin were prepared using a 100 g polymer to 33 mL monomer ratio—group I (conventional polymer), group II (polymer‐reinforced with 2.5% nanocellulose), and group III (polymer‐reinforced with 5% nanocellulose). The acrylic resin was packed at the dough stage, and trial closure was performed to eliminate excess material. Final closure was completed followed by bench curing. The flask was immersed in water, gradually heating it to 72°C over 60 min, and then increasing the temperature to 100°C for an additional 30 min. The flask was allowed to cool to room temperature, deflasked, and the specimen retrieved. All the specimens were stored in 37°C distilled water for 7 days prior to flexural strength testing [14].

#### 2.4.2. Testing Methodology

Test specimens were fabricated with dimensions of 65 mm × 10 mm × 3.3 mm in accordance with ISO 20795‐1:2013 specifications for denture base polymers [15].

#### 2.4.3. Testing Methodology

Flexural strength was evaluated using a three‐point bending test in accordance with the ISO 20795‐1:2013 standards. Testing was performed using a universal testing machine (Kalpak Instruments and Controls, Pune, India). Each specimen was positioned on two supporting wedges with a span length of 50 mm. The load was applied at the midpoint of the specimen using a loading wedge at a constant crosshead speed of 5 mm/min until fracture occurred.

**Figure 1:**
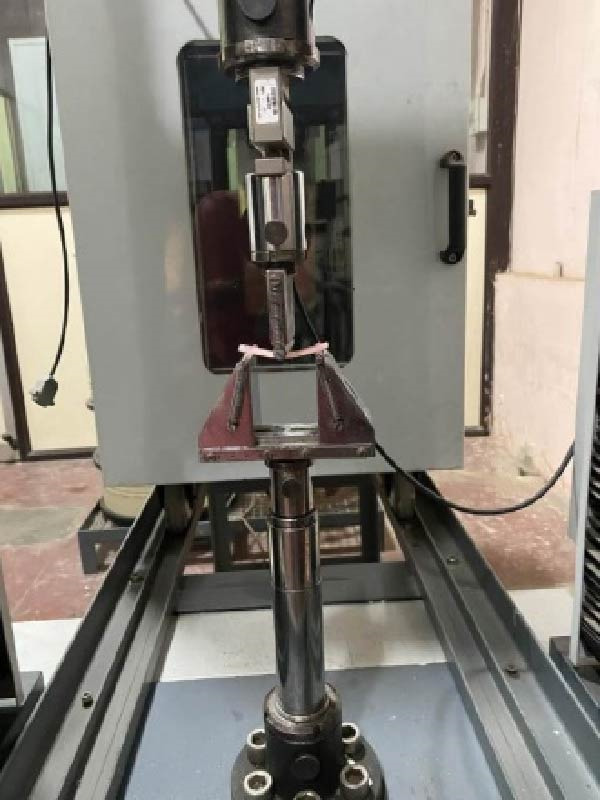
Testing of flexural strength in universal testing machine.

The maximum load at fracture (N) was recorded using a computerized data acquisition system, and the corresponding load–deflection curves were obtained. All specimens were aligned centrally to ensure a uniform stress distribution during testing (Figure 1).

Flexural strength was calculated using the following formula:

Flexural strength=32Pl/bd2,

where *P* is the maximum load (force) at the fracture point (N). *l* is the length of the support span = 50 mm. *b* is the width (mm) of the specimen = 10 mm. *d* is the thickness (mm) of the specimen = 3.3 mm. Flexural strength was expressed in newtons per millimeter square.

### 2.5. Assessment of Impact Strength

#### 2.5.1. Fabrication of Sample

Wax patterns were created using a metal mold of dimension 50 mm × 6 mm × 4 mm with a 1.2 mm notch in the middle prepared according to ISO 1567:1999. The samples were acrylized using the same protocol for the fabrication of specimens for testing flexural strength. The samples were labeled at both ends before testing.

#### 2.5.2. Testing Methodology

Impact strength was evaluated using an Izod pendulum impact testing machine (International Equipment, Mumbai, India) in accordance with ASTM D256. The specimens were centrally notched to obtain a standardized V‐notch. Each specimen was mounted vertically in the testing apparatus, with the notch facing the pendulum striker.

**Figure 2:**
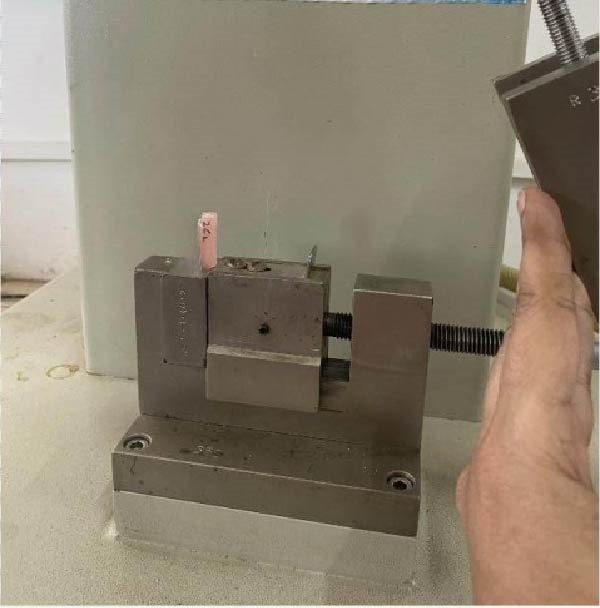
Testing of impact strength.

A pendulum hammer with a capacity of 5.4 J was released from a fixed initial angle of 150° to strike the specimen at the notch. The energy absorbed by the specimen at the point of fracture was recorded from the digital display of the machine. Impact strength was calculated by dividing the absorbed energy by the cross‐sectional area at the notch and expressed in kJ/m^2^ (Figure 2).

The impact strength was calculated using the following formula:

IS=Energy absorbed/effective width× thickness×1000,

where IS is the impact strength in kJ/m^2^, energy absorbed is the net energy absorbed in Joules, effective width is total width minus notch depth (6 mm − 1.2 mm = 4.8 mm), and thickness is 4 mm.

### 2.6. Assessment of Antimicrobial Property

#### 2.6.1. Fabrication of Specimen

Wax patterns were created using metal molds of dimension measuring 6 mm in diameter. The specimens were acrylized using the procedure mentioned earlier. The specimens were kept in distilled water at 37°C for 1 week, after which well diffusion assay was carried out.

#### 2.6.2. Testing Methodology

**Figure 3:**
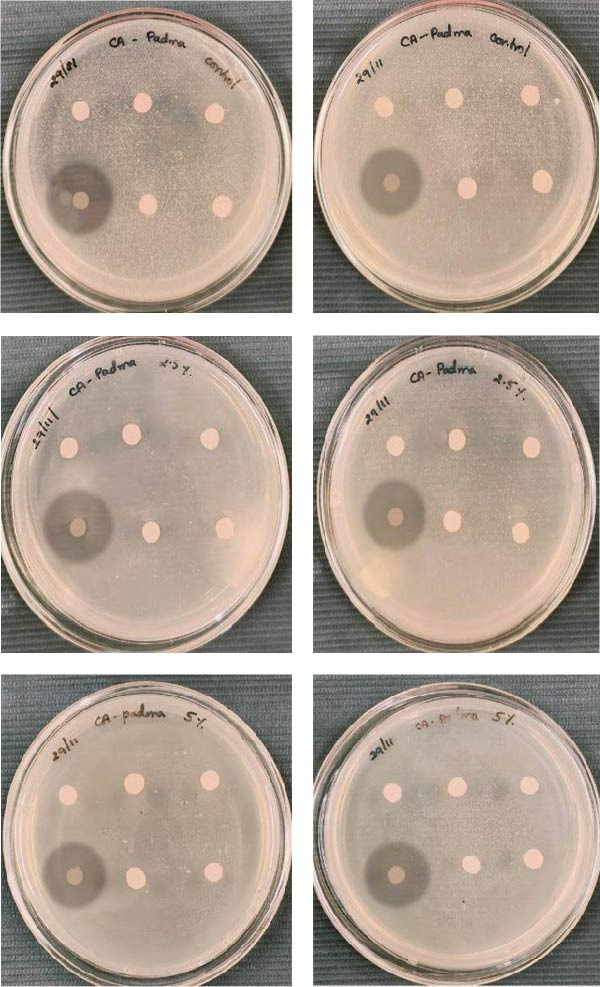
No zone of inhibition was observed in the control group, 2.5%, and 5% nanocellulose group, except in the positive control.

i. Fungal cultures and growth condition: *Candida albicans* ATCC90028 was grown in potato dextrose broth for 2–3 days at 300°C under constant shaking (150 rpm).
ii. Assay protocol‐well diffusion assay: Potato dextrose agar (PDA) medium was poured into sterile petri plates and left to solidify. Fungal pathogen (0.5 OD at 600 nm) was spread on the media using sterile swabs. After 3–4 min of drying, a specimen (disc) was placed on the plate at a specific region using sterile forceps. The plates were incubated at 30°C for 1–2 days and examined for the presence of inhibition zones. Antibiotic fluconazole (1 mg/mL) was used as a positive control (Figure 3).

All the data obtained from flexural strength, impact strength, and antimicrobial property testing were collated and analyzed using SPSS software.

### 2.7. Statistical Analysis

Data were analyzed using SPSS software (version 25, IBM Corp., USA). One‐way analysis of variance (ANOVA) was used to compare mean values among groups, followed by the Bonferroni post hoc test for pairwise comparisons. A *p*‐value < 0.05 was considered statistically significant.

## 3. Results

### 3.1. Flexural Strength

The polymer reinforced with the 5% nanocellulose group showed the highest mean flexural strength (60.82 MPa) but with the greatest variability, while the control group had the lowest strength (53.66 MPa) and most consistent results. Overall, the strength increased with percentage, with the combined mean at 56.54 MPa (Table 1).

**Table 1:** Descriptive statistical evaluation of flexural strength among the three groups.

Groups	*N*	Mean	Std. deviation	Std. error	95% Confidence interval for mean		Minimum	Maximum
					Lower bound	Upper bound		
2.5%	10	55.1560	7.52707	2.38027	49.7715	60.5405	43.75	67.90
5%	10	60.8150	7.83235	2.47681	55.2121	66.4179	51.20	69.08
Control	10	53.6600	6.93734	2.19378	48.6973	58.6227	41.55	67.91
Total	30	56.5437	7.83453	1.43038	53.6182	59.4691	41.55	69.08

ANOVA showed no significant difference in flexural strength among the 2.5%, 5%, and control groups (*F* = 2.572, *p* = 0.095). The variation observed was likely due to chance, indicating insufficient evidence to reject the null hypothesis (Table 2).

**Table 2:** ANOVA one‐way of flexural strength.

Groups comparison	Sum of squares	df	Mean square	*F*	Sig. (*p* < 0.05)
Between groups	284.854	2	142.427	2.572	0.095 (NS)
Within groups	1495.163	27	55.376		
Total	1780.018	29	—		

Abbreviation: NS, not significant.

The Bonferroni post hoc test showed no statistically significant differences in flexural strength among the 2.5%, 5%, and control groups. All *p*‐values were above 0.05, and confidence intervals included zero, indicating that the observed differences were likely due to random variation. Although the 5% group had the highest average flexural strength, followed by the 2.5% and control groups, these differences were not statistically significant, aligning with the ANOVA results (Table 3).

**Table 3:** Bonferroni post hoc test of flexural strength.

(*I*) Groups	(*J*) Groups	Mean difference (*I* − *J*)	Std. error	Sig.	95% Confidence interval	
					Lower bound	Upper bound
2.5%	5%	−5.65900	3.32795	0.302 (NS)	−14.1535	2.8355
	Control	1.49600	3.32795	1.000 (NS)	−6.9985	9.9905
5%	2.5%	5.65900	3.32795	0.302 (NS)	−2.8355	14.1535
	Control	7.15500	3.32795	0.122 (NS)	−1.3395	15.6495
Control	2.5%	−1.49600	3.32795	1.000 (NS)	−9.9905	6.9985
	5%	−7.15500	3.32795	0.122 (NS)	−15.6495	1.3395

*Note:* The mean difference is significant at the 0.05 level.

Abbreviation: NS, not significant.

### 3.2. Impact Strength

The 5% group had the highest mean impact strength (12.66 KJ/m^2^) and greatest variability, while the control group had the lowest mean (5.19 KJ/m^2^) and highest consistency. The 2.5% group showed intermediate values. Overall, impact strength varied notably across groups, with an overall mean of 8.43 KJ/m^2^ (Table 4).

**Table 4:** Descriptive statistics for impact strength.

Groups	*N*	Mean	Std. deviation	Std. error	95% Confidence interval for mean		Minimum	Maximum
					Lower bound	Upper bound		
2.5%	10	7.4381	0.80903	0.25584	6.8593	8.0168	5.21	7.94
5%	10	12.6612	2.98553	0.94411	10.5254	14.7969	10.42	18.23
Control	10	5.1936	0.08355	0.02642	5.1338	5.2534	5.10	5.34
Total	30	8.4309	3.61883	0.66070	7.0797	9.7822	5.10	18.23

ANOVA results showed a significant difference in impact strength among the three groups (*F* = 45.996, *p*  < 0.001). The variation was mainly due to differences between group means (SS = 293.608), confirming that concentration significantly affected the impact strength. Post hoc analysis is needed to identify which groups differ (Table 5).

**Table 5:** ANOVA one‐way for impact strength.

Groups comparison	Sum of squares	df	Mean square	*F*	Sig. (*p* < 0.05)
Between groups	293.608	2	146.804	45.996	0.001 (HS)
Within groups	86.174	27	3.192		
Total	379.782	29	—		

Abbreviation: HS, highly significant.

The Bonferroni post hoc test (Table 6) confirmed significant differences in the impact strength among all groups. The 5% group had significantly higher impact strength than both the 2.5% and control groups (*p* = 0.001), and the 2.5% group was significantly stronger than the control (*p* = 0.027). All confidence intervals excluded zero, validating these differences. Overall, impact strength ranked highest in the 5% group, followed by 2.5%, and lowest in the control group.

**Table 6:** Bonferroni post hoc test for impact strength.

(*I*) Groups	(*J*) Groups	Mean difference (*I* − *J*)	Std. error	Sig.	95% Confidence interval	
					Lower bound	Upper bound
2.5%	5%	−5.22307	0.79895	0.001 (HS)	−7.2624	−3.1838
	Control	2.24447	0.79895	0.027 (S)	0.2052	4.2838
5%	2.5%	5.22307	0.79895	0.001 (HS)	3.1838	7.2624
	Control	7.46754	0.79895	0.001 (HS)	5.4282	9.5068
Control	2.5%	−2.24447	0.79895	0.027 (S)	−4.2838	‐0.2052
	5%	−7.46754	0.79895	0.001 (HS)	−9.5068	−5.4282

*Note:* The mean difference is significant at the 0.05 level.

Abbreviations: HS, highly significant; S, significant.

### 3.3. Antimicrobial Property

The study found no antifungal activity in the test specimens against *Candida albicans* (ATCC90028), as no zone of inhibition was observed on PDA plates. A 20 mm zone from the fluconazole control confirmed the assay’s validity. Thus, the samples lacked effective antifungal compounds under the tested conditions.

## 4. Discussion

PMMA is widely used in dentures due to its low cost, biocompatibility, ease of processing, and esthetic appeal. However, heat‐cured acrylic resin has limitations, including low flexural strength, impact resistance, and antimicrobial properties, which can reduce the clinical lifespan of prostheses [13]. The present study compared the flexural strength, impact strength, and antimicrobial properties of nanocellulose‐reinforced heat‐cured PMMA with conventional heat‐cured PMMA. Nanocellulose, a sustainable, renewable plant‐based material, offers benefits like high mechanical strength, flexibility, biodegradability, and biocompatibility. It enhances polymer matrices, extending the lifespan of acrylic materials, and exhibits excellent optical performance. Nanocellulose is increasingly used in green packaging, filtration, and biomedical applications, particularly for its antimicrobial properties.

Cellulose nanofibers (CNF) can bind and deliver antimicrobial peptides like nisin, enhancing the antimicrobial activity of biopolymers used in food packaging, such as polypropylene, polyethylene, and polylactic acid [14].

Carbon nanofiber cellulose (CNC) exhibits antimicrobial properties, high moisture retention, and superior mechanical stability, making them ideal for wound dressings, drug delivery, and tissue engineering. Additionally, cellulose nanocomposites are used in antimicrobial coatings for paper and board, offering both reinforcement and antimicrobial functionality. These properties highlight nanocellulose’s potential for innovative antimicrobial applications in the biomedical and packaging sector [15]. Hence, due to multiple benefits of nanocellulose, it was considered as a reinforcement material for PMMA in the present study.

Nanocellulose fibers derived from cotton were used at 2.5% and 5% concentrations in the study, based on research by Sagadevan K et al. [13] on cellulose nanoparticle‐reinforced PMMA. The nanocellulose was incorporated into heat‐cured acrylic resin using the geometric dilution method, as per Sagadevan K et al.’s [13] approach for uniform distribution of zirconium dioxide and cellulose.

Geometric dilution is a mixing process where nanoparticles and PMMA are measured and homogenized to prevent agglomeration, which can weaken the material. This method ensures uniform distribution, enhancing the mechanical properties of PMMA, such as fracture resistance and flexural strength, by facilitating effective stress transfer. It also reduces variability, providing consistent and reproducible results, and is crucial for optimizing the interaction between nanoparticles and polymers to improve PMMA denture bases.

### 4.1. Flexural Strength

The study found that nanocellulose reinforcement had minimal impact on flexural strength, with the 5% group achieving the highest mean strength (60.815 MPa), followed by 2.5% (55.156 MPa) and the control group (53.660 MPa). However, ANOVA analysis (*p* = 0.095) indicated no statistically significant differences. This contrasts with a previous study by Sagadevan K et al. [13], which showed significant improvements in flexural strength (71.47 MPa) when nanocellulose and zirconia were used. The difference suggests that the source of nanocellulose may affect the reinforcement’s effectiveness [13].

Nanocellulose may improve impact resistance more than stiffness, which affects flexural properties. Flexural strength, influenced by matrix stiffness and interfacial adhesion, may not be significantly enhanced by nanocellulose if it fails to form a strong interface with the polymer matrix.

### 4.2. Impact Strength

The study showed that nanocellulose significantly improved impact strength, with the 5% group achieving the highest mean (12.6612 MPa), followed by 2.5% (7.4381 MPa) and control (5.1936 MPa). This differs from previous research by Senbagavalli and Sagadevan et al. [13], which may be due to differences in the source of nanocellulose affecting the impact strength of the reinforced material.

The study showed that increasing nanocellulose concentrations improved the impact strength of PMMA, aligning with Hasran et al. [16] findings, where higher nanocellulose concentrations (1%–5%) in heat‐cured acrylic resin boosted impact strength. This enhancement is attributed to nanocellulose’s large surface area and strong hydrogen bonding, which improves stress distribution and crack resistance. The 5% nanocellulose group exhibited the highest impact strength, confirming that higher filler concentrations improve acrylic resin’s impact resistance [16].

The study by Fadhel and Safi [17] found a significant increase in impact strength with 0.5% and 1% nanocellulose concentrations, but no significant increase at 1.5% and 2%. This contradicts the present study’s findings, where higher nanocellulose concentrations improved impact strength. The discrepancy may be due to better dispersion of nanocellulose fibers in the polymer compared to their incorporation into the monomer [17]. Proper dispersion of nanoparticles provided better stress distribution and energy absorption, which acts as reinforcement bridges within the acrylic matrix.

### 4.3. Antimicrobial Property

The antimicrobial test showed no antifungal activity against *Candida albicans* in the nanocellulose‐reinforced acrylic resin specimens, as no inhibition zone was observed. This may be due to the lack of inherent antifungal properties in nanocellulose or its insufficient concentration in the resin. A positive control (fluconazole) confirmed the assay’s validity, producing a 20 mm inhibition zone. Nanocellulose, being chemically inert and lacking biocidal groups, does not exhibit antimicrobial effects, aligning with previous studies [18].

A review on nanocellulose‐based antimicrobials highlights that while nanocellulose has potential as a scaffold due to its porous structure and hydroxyl groups, it needs surface modification or additives to exhibit antifungal properties. Bacterial nanocellulose (BNC) can limit microbial attachment and biofilm formation due to its moisture‐absorbing properties, but it does not inherently possess antimicrobial activity. For effective antimicrobial effects, nanocellulose must be functionalized with agents like silver or TEMPO oxidation. In its unmodified form within heat‐cured acrylic resin, nanocellulose acts as an inert filler and does not inhibit *Candida albicans* growth, as its levels are too low and it is trapped in the resin, especially after polishing.

The study’s limitations include being conducted under laboratory conditions, which may differ from in vivo scenarios. Despite using geometric dilution, nanoparticle agglomeration could still occur, creating weak zones and inconsistencies. The sample size may have affected the detection of statistically significant differences. Additionally, only two concentrations of nanocellulose (2.5% and 5%) were tested, and the effects of higher or lower concentrations were not explored. Although geometric dilution was used to improve dispersion, the absence of advanced techniques (e.g., ultrasonication) and microscopic validation may have allowed some nanoparticle agglomeration, potentially affecting the mechanical properties and contributing to result variability. Future studies are recommended to include SEM or similar analyses.

## 5. Conclusions

Within the limitations of this in vitro study, reinforcement of heat‐cured acrylic resin with nanocellulose at 2.5% and 5% concentrations showed an improvement in flexural strength compared to the control, but the differences were not statistically significant. Impact strength increased significantly, with the 5% group exhibiting superior performance over both the 2.5% and control groups. The results indicate that 5% nanocellulose‐reinforced acrylic resin effectively enhances the impact resistance of heat‐cured acrylic resin. Clinically, this suggests a potential reduction in denture fracture risk during function or accidental handling, thereby improving the prosthesis durability. However, as no antimicrobial activity was observed, nanocellulose reinforcement cannot be relied upon for microbial control, and further in vivo studies are necessary.

## Funding

This study was supported by the JSSAHER Research Fund.

## Conflicts of Interest

The authors declare no conflicts of interest.

## Data Availability

The data that support the findings of this study are available from the corresponding author upon reasonable request.
